# Parent substance use and child‐to‐parent violence: A brief report

**DOI:** 10.1111/dar.14031

**Published:** 2025-03-03

**Authors:** Ashlee Curtis, David Skvarc, Noa Brittain, Peter G. Miller, Richelle Mayshak, Travis Harries

**Affiliations:** ^1^ School of Psychology Deakin University Geelong Australia

**Keywords:** aggression, child to parent violence, substance use

## Abstract

**Introduction:**

Substance use has been associated with child‐to‐parent violence (CPV), yet little is known about the contributing factors. This study investigated the association between parental substance use and substance involved child to parent violence (SU‐CPV; i.e., the young person is influenced by a substance), and whether this association was unique to SU‐CPV compared to other co‐occurring functions of CPV (proactive/instrumental, reactive/response to threat, affective/emotion‐related).

**Methods:**

One hundred and nineteen caregivers experiencing abuse‐level CPV from a young person (97% female; aged 27–78 years; *M*age = 48.4, SDage = 7.34) completed an online survey assessing their own, and the young person's substance use, and the young person's use of CPV.

**Results:**

Multivariate multiple linear regression analyses demonstrated parental alcohol use was significantly positively associated with SU‐CPV (*b* = 0.29, *p* = 0.002), but not other CPV functions. There was no significant association between parental drug use and SU‐CPV.

**Discussion and Conclusions**

Parental alcohol use was associated with increased likelihood of SU‐CPV, suggesting parental behaviours may influence the young person's behaviour, and this may be exacerbated by the disinhibiting impact of alcohol and unpredictability within the home environment. Parent alcohol use may also be a coping strategy for experiences of SU‐CPV. The lack of association with parent drug use may be due to few parents reporting drug use and the varied impact of differing drug types on the home environment. Substance use is a key contributing factor for aggressive and violent behaviour, highlighting the role of prevention and intervention efforts encompassing the broader family.

## 1 INTRODUCTION

Child‐to‐parent violence (CPV) is a form of family violence including physical or non‐physical aggression towards a parent or caregiver by a young person under their care [1]. In Australia, 8.4% of all family violence incidents are young people using violence against parents [2], indicating a need to understand the contributing factors such as substance use. CPV is frequently associated with substance use [3]; in Victoria, 35% of young people who were reported to police for CPV self‐reported a substance use issue, and police identified that 28% were likely to have been substance affected at the time of the reported incident [3]. Understanding the association between substance use and CPV is essential to informing targeted prevention and intervention efforts (the majority of young people entering some CPV intervention programs have substance use problems; [4]), and disrupting the intergenerational impact of both substance use and violence [5, 6].

Substance‐involved CPV (SU‐CPV) is not yet well understood. Indeed, instances of CPV occurring while the young person is substance‐affected have previously been excluded from consensus definitions of CPV [7], despite studies suggesting that the young person's substance use (both alcohol and other drug use) and their use of violence against the parent are associated [8, 9]. One explanation for this association is Alcohol Myopia theory, whereby alcohol (or other substance) intoxication narrows attentional focus to salient cues in the environment, while also reducing informational processing capacity [10]. Intoxicated individuals are thus more likely to respond to provocative cues over non‐provocative or ambiguous cues [10]. Further, alcohol (or other substance) exposure may be enough to increase aggressive responses to ambiguous cues [11], particularly if the young person already has a tendency to interpret ambiguous cues as hostile [12]. As such young persons who are intoxicated may be more likely to respond to perceived provocation with aggression, and this process may be enhanced where the young person is in an environment where the parent is also using substances and is similarly cognitively impacted. The impact of both members of the dyad being substance‐affected may create a more hostile or unpredictable environment in which conflict is more likely. For instance, CPV may arise due to arguments about the young person's substance use (e.g., the young person's perception that the parents are attempting to control their behaviour), during intoxication or drug use, or while withdrawing/abstaining [3, 9]. Alternatively, the unpredictability of the environment resulting from the dyadic substance use may lead to high levels of emotion dysregulation within the family unit, and any difficulties in managing such dysregulation by the parents are modelled by the young person [13]. Such dysregulation may lead to the use of violence by the young person as a way to gain control over the unpredictable environment or associated inconsistency from the caregiver [14].

CPV has been argued to serve three functions: Proactive which is instrumental and planned, reactive which occurs in response to a real or perceived threat (e.g., parental use of corporal punishment [15]), and affective occurring due to internal fear or frustration [16]. It is likely that SU‐CPV overlaps with, and contributes to, each of these functions; proactive CPV may be utilised to obtain money to purchase substances, reactive CPV may be a result of the substance myopic effect, and affective CPV may occur during times of withdrawal of abstinence. This cross‐functional nature of SU‐CPV necessitates targeted prevention and intervention efforts, particularly given the known exacerbating impact of substance use on aggression [10, 17]. Therefore, the current study explored the association between parental substance use and SU‐CPV, and whether this association was unique to SU‐CPV compared to other co‐occurring functions of CPV.

## 2 METHOD

### 2.1 Participants

The study involved 119 Australian caregivers experiencing CPV, aged between 27 and 78 years (*M* = 48.4, SD = 7.34; 97% female), who were recruited via Facebook and Instagram advertisements for a study on ‘child–parent’ conflict. Most participants were a biological parent of the young person (87%; 5.9% adoptive, step or foster parent; 0.8% legal guardian). Participants reported on young people aged 12–24 years (*M* = 15.94, SD = 3.25; 40% female) and were excluded if they were not experiencing abuse‐level CPV (i.e., score <16 on the Abusive Behaviour by Children—Indices; [18]) or if the young person was aged less than 12 years. An estimate of family socioeconomic status was identified using the deciles of socioeconomic disadvantage based on the respondent's postcode (where decile 1 contains the most disadvantaged areas and decile 10 contains the most advantaged areas). Using this method, 61.3% of the sample were in the bottom 50% of disadvantage (deciles 1–5), and the average socioeconomic decile of the sample was 5.05 (SD = 2.62).

### 2.2 Measures

#### 2.2.1 Child‐to‐parent violence

CPV form was measured using the Abusive Behaviour by Children—Indices [18], which considers cultural norms and severity and frequency of aggressive behaviour over the past 12 months in order to distinguish between abusive and non‐abusive CPV. A score of 16 or higher indicates CPV (see Simmons et al. [18] for a full description).

CPV function was assessed using the CPV‐F [19], which measures affective (e.g., ‘when the young person is aggressive … they appear anxious, frightened, or scared’; α = 0.62), reactive (‘The young person immediately seeks retaliation against me by being violent or aggressive’; α = 0.82) and proactive (‘The young person is devious’; α = 0.94). CPV across 18‐items measured on a five‐point scale (0 = never, 4 = almost always). Subscale scores were the mean response across items.

The substance‐involved nature of CPV (SU‐CPV) was measured using three items on a five‐point scale (0 = never, 4 = almost always): ‘When the young person is being violent or aggressive toward me …’ 1. ‘… they are coming down from drug or alcohol use’, 2. ‘… they had been withholding or abstaining from their usual drug or alcohol use’, and 3. ‘… they are under the influence of drugs or alcohol’. The average scores on these items were calculated to create a latent variable; reliability was excellent (α = 0.92).

#### 2.2.2 Parent substance use

The Alcohol Use Disorders Identification Test (AUDIT‐C [20]) and the Drug Use Disorders Identification Test [21] were used to measure parent substance use. The AUDIT‐C measured problematic alcohol consumption over the past 12 months on a scale of 0 to 4 using three items; problematic drinking was defined as a score of ≥4 for males and a score of ≥3 for females. The Drug Use Disorders Identification Test comprised 11 items measuring problematic drug use over the past 12 months; the first nine were scored on a five‐point Likert scale (0 to 4), with two items scored on a three‐point Likert scale (0, 2, 6), problematic drug use was defined as a score of ≥6 for males and a score of ≥2 for females.

### 2.3 Procedure

Ethics approval was attained from the Deakin University Human Research Ethics Committee (ID: 2020–227). Participants completed an online survey after being shown a Plain Language Statement and providing informed consent. Based on a‐priori power analyses, a minimum sample of 114 participants was needed to detect a moderate effect (*F* ^2^ = 0.10) at 80% power (α of 0.05). Study materials are not available due to ethical restrictions.

## 3 RESULTS

Descriptive statistics and correlations between key variables are presented in Table 1. Overall, problematic drug use was less common than risky alcohol use. There were significant positive correlations between SU‐CPV and parent AUDIT‐C scores and the age of the young person. SU‐CPV was also positively corelated with proactive and reactive CPV. Child gender was only weakly positively associated with proactive CPV and, as such, we opted not to include it in further analyses.

**TABLE 1:** Descriptive statistics and correlations for key variables.

	Descriptives			Correlations					
	*N* (%)	Mean	Gender (YP)	Age (YP)	AUDIT‐C (P)	DUDIT (P)	Reactive (YP)	Proactive (YP)	Affective (YP)
Age (YP)		15.94	−0.07						
AUDIT‐C (P)	25 (21%)		−0.02	0.14					
DUDIT (P)	7 (6%)		−0.04	0.21^a^	0.10				
Reactive (YP)		1.82	0.12	−0.05	0.02	0.00			
Proactive (YP)		1.58	0.24^b^	−0.03	−0.06	0.03	0.77^b^		
Affective (YP)		2.19	0.05	0.07	−0.12	−0.16	0.11	0.03	
SU‐CPV (YP)	29 (24%)		0.09	0.39^b^	0.35^b^	0.15	0.24^a^	0.22^a^	0.00

*Note*: Gender reference category is male.

Abbreviations: AUDIT, Alcohol Use Disorders Identification Test; DUDIT, Drug Use Disorders Identification Test; P, parent; SU‐CPV, substance involved child‐to‐parent violence; YP, young person.

^a^ Correlation is significant at the 0.05 level (2‐tailed).

^b^ Correlation is significant at the 0.01 level (2‐tailed).

### 3.1 Inferential statistics

We used multivariate multiple linear regression to estimate the unique relationship between SU‐CPV (alongside other functions of CPV) and parent alcohol and drug use, while controlling for young person age. This was necessary as the age range in our sample crossed over the legal drinking age in Australia. SU‐CPV is relatively rare and so not normally distributed. As such, we dichotomised SU‐CPV to indicate binary presence or absence. Both the age of the young person (*F* = 4.41, *p* = 0.001) and parent alcohol use (*F* = 2.90, *p* = 0.018) made significant contributions to the overall model. Specifically, the results suggest that parent alcohol use, but not drug use is positively associated with SU‐CPV, but not other functions (Table 2).

**TABLE 2:** Regression analysis for parent alcohol use, parent drug use, and the functions of CPV, controlling for age.

Source	Dependent variable	b	F	Sig.	95% CI		Partial eta squared	OR
					Lower	Upper		
Age (YP)	Reactive CPV	0.00	0.00	0.962	−0.056	0.059	0.00	
	Proactive CPV	0.00	0.00	0.981	−0.064	0.065	0.00	
	Affective CPV	0.03	0.87	0.353	−0.031	0.087	0.01	
	SU‐CPV	0.04	12.00	**<0.001**	**0.019**	**0.069**	**0.12**	1.04
Parent alcohol use	Reactive CPV	0.12	0.35	0.558	−0.285	0.524	0.00	
	Proactive CPV	−0.08	0.12	0.728	−0.531	0.372	0.00	
	Affective CPV	−0.24	1.33	0.253	−0.652	0.174	0.02	
	SU‐CPV	0.29	10.68	**0.002**	**0.114**	**0.467**	**0.11**	1.34
Parent drug use	Reactive CPV	−0.04	0.01	0.909	−0.722	0.644	0.00	
	Proactive CPV	0.11	0.08	0.775	−0.653	0.872	0.00	
	Affective CPV	−0.44	1.54	0.218	−1.133	0.262	0.02	
	SU‐CPV	0.06	0.15	0.697	−0.240	0.357	0.00	

Abbreviations: CI, confidence interval; CPV, child‐to‐parent violence OR, odds ratio; SU‐CPV, substance involved child‐to‐parent violence; YP, young person.

## 4 DISCUSSION

The current study investigated the association between parental substance use and SU‐CPV, and whether this association was unique to SU‐CPV compared to other co‐occurring functions of CPV. Only parental alcohol use was significantly positively associated with SU‐CPV, but not with any other functions of CPV. There was no significant association between parental drug use and SU‐CPV.

Social learning theory predicts that parental alcohol use is more likely to be associated with SU‐CPV. Parental substance use is one of the most common predictors of child substance use [13, 22], with one explanation for this being observation and imitation of their parents [23]. This same pathway holds for aggressive and violent behaviour, whereby young persons may learn that aggression and violence are appropriate responses to conflict within the family [24]. This is likely to be exacerbated by the myopic effect of alcohol, whereby it not only reduces inhibition, but also increases the likelihood of an aggressive response to an ambiguous or non‐provocative cue [10]. Further to this, the unpredictability of the environment created by parental alcohol use may lead to the young person using substances as a coping mechanism for such an environment which may lead to a myopic impact, or to higher levels of emotion dysregulation among both the parent and the young person, creating further opportunities for conflict as a means to gain control over both the dysregulation and the unpredictable environment. It may also be that parent alcohol use is a coping mechanism for experiences of SU‐CPV.

We expected that parent substance use may be associated with reactive CPV, given that alcohol use is associated with harsh parenting [25] that may trigger a reaction from the child, however, we did not find this. It may be that the average level of substance use was not high enough in our sample to observe this, but high enough still for it to be modelled by the young person and for SU‐CPV to emerge. The lack of relationship between SU‐CPV and parent drug use was also unexpected. However, this may have occurred due to the small number of parents reporting drug use (*n* = 7) and the variation in impact of different drug types on behaviour within the home (i.e., sedatives/opioids vs. amphetamines). Indeed, the small number of parents reporting drug use limited our ability to conduct meaningful analyses, and this likely resulted from our small sample size. It is important to note that our sample was restricted to only parents who were experiencing abuse‐levels of CPV within Australia who responded to social media advertisements. Expanding beyond Australia, using a variety of recruitment methods, and including a broader definition of CPV may provide more insight into the association between parent drug use, parent substance use more broadly, and SU‐CPV.

This study demonstrates a link between parent substance use and SU‐CPV and further argues for the need to include instances of substance‐related aggression towards a parent in general definitions of CPV. However, this study does not provide insight into how SU‐CPV emerges, the key contextual and historical variables which may be important, or what influences the behaviour continuing. Such knowledge is necessary to ensure prevention and intervention approaches can be targeted appropriately, and the young person can be best supported not to use violence. One approach to this which may provide necessary insight is the collection of ‘in the moment’ experiences of CPV where parents and the young person are using substances and there is a resultant incident of CPV. This may be undertaken using methods such as a daily diary, Ecological Momentary Assessment, or critical incident technique which allow for collection of data specific to an incident when the information around the precipitating and perpetuating factors are easily recalled. This may also allow for factors such as poor parental monitoring [16], childhood maltreatment, intergenerational trauma [26], and co‐morbid mental health concerns for the young person and parent [16] to be integrated into a broader understanding of SU‐CPV. It may also allow for us to capture experiences of SU‐CPV from the perspective of both parents, given the current study (consistent with the CPV literature more broadly) comprised primarily female caregiver respondents. Despite the clear need for larger, more sophisticated research into this issue, this finding reinforces the importance of substance use for some young people who engage in violent behaviour.

## AUTHOR CONTRIBUTIONS

Each author certifies that their contribution to this work meets the standards of the International Committee of Medical Journal Editors.

## CONFLICT OF INTEREST STATEMENT

The authors have no declarations of interest.

## Data Availability

The data that support the findings of this study are available on request from the corresponding author. The data are not publicly available due to privacy or ethical restrictions.
